# Hydrogen-Deuterium Exchange Mass-Spectrometry of Secondary Active Transporters: From Structural Dynamics to Molecular Mechanisms

**DOI:** 10.3389/fphar.2020.00070

**Published:** 2020-02-19

**Authors:** Moshe Giladi, Daniel Khananshvili

**Affiliations:** ^1^Department of Physiology and Pharmacology, Sackler Faculty of Medicine, Tel Aviv University, Tel Aviv, Israel; ^2^Tel Aviv Sourasky Medical Center, Tel Aviv University, Tel Aviv, Israel

**Keywords:** mass spectrometry, hydrogen-deuterium exchanger, secondary transport, membrane proteins, structural dynamics

## Abstract

Membrane transporters allow the selective transport of otherwise poorly permeable solutes across the cell membrane and thus, play a key role in maintaining cellular homeostasis in all kingdoms of life. Importantly, these proteins also serve as important drug targets. Over the last decades, major progress in structural biology methods has elucidated important structure-function relationships in membrane transporters. However, structures obtained using methods such as X-ray crystallography and high-resolution cryogenic electron microscopy merely provide static snapshots of an intrinsically dynamic, multi-step transport process. Therefore, there is a growing need for developing new experimental approaches capable of exploiting the data obtained from the high-resolution snapshots in order to investigate the dynamic features of membrane proteins. Here, we present the basic principles of hydrogen-deuterium exchange mass-spectrometry (HDX-MS) and recent advancements in its use to study membrane transporters. In HDX-MS experiments, minute amounts of a protein sample can be used to investigate its structural dynamics under native conditions, without the need for chemical labelling and with practically no limit on the protein size. Thus, HDX-MS is instrumental for resolving the structure-dynamic landscapes of membrane proteins in their apo (ligand-free) and ligand-bound forms, shedding light on the molecular mechanism underlying the transport process and drug binding.

## Introduction

Membrane proteins participate in fundamental physiological events in every biological system from bacteria to humans. >50% of marketed drugs target membrane proteins, whereas the pharmacological targeting of many other membrane proteins is considered potentially beneficial in numerous biomedical applications (Overington et al., 2006; Yıldırım et al., 2007; Arinaminpathy et al., 2009). However, the molecular mechanisms underlying their function and regulation remain largely unknown due to the lack of structural information. Indeed, while membrane proteins account for ~26% of the human proteome, their structures represent only ∼2% of the structures in the Protein Data Bank (Fagerberg et al., 2010; Pandey et al., 2016). Several obstacles hamper the structural investigation of membrane proteins, requiring the development of state-of-the-art technologies for elucidating their molecular architectures (Pandey et al., 2016; Masson et al., 2017; Hagn et al., 2018; Ravula and Ramamoorthy, 2019; Redhair et al., 2019). The major obstacles are that their overexpression and purification and structural studies by most techniques (e.g., X-ray crystallography) remain limited in many cases. These obstacle consequently hamper the rational development of drug candidates targeting disease-related membrane proteins (Vinothkumar and Henderson, 2010; Lounnas et al., 2013; Marciano et al., 2014).

Secondary transporters are membrane-bound proteins that selectively catalyze the movement of poorly permeable solutes (e.g., ions, neurotransmitters, drugs) across the cell membrane, supporting diverse cellular functions in health and disease. Secondary transporters comply with the alternating access paradigm, according to which the protein ligand-binding pocket becomes alternatively accessible at opposite sides of the membrane by adopting the inward-facing (IF) and outward-facing (OF) states in succession (Jardetzky, 1966; Forrest et al., 2011). Recent breakthroughs in the structural biology of membrane proteins provided a wealth of structures of membrane-bound transporters in different conformational states (Drew and Boudker, 2016; Bai et al., 2017), providing insights into their transport and solute recognition mechanisms. However, they provide static snapshots of an inherently multi-step process (Seeger, 2018). Thus, there is a growing need for developing new approaches to investigate membrane proteins dynamics (Konermann et al., 2011; Smith et al., 2012; Sim et al., 2017; Mandala et al., 2018; Burke, 2019). These include a combination of molecular dynamics (MD) simulations (Roux et al., 2011; Zdravkovic et al., 2012; Zhekova et al., 2016; Zhekova et al., 2017; Harpole and Delemotte, 2018) with advanced experimental techniques, including spectroscopic techniques and single-particle cryogenic electron microscopy (Smith et al., 2012; Pandey et al., 2016; Castell et al., 2018; Hagn et al., 2018; Ravula and Ramamoorthy, 2019; Sun and Gennis, 2019).

Hydrogen-deuterium exchange mass-spectrometry (HDX-MS) gained attention in recent years for studying membrane proteins dynamics, even though it has been used for decades to characterize soluble proteins (Konermann et al., 2011; Vadas et al., 2017). HDX-MS monitors time-dependent exchange of solvent D_2_O with proteins backbone amide hydrogens under native conditions. The deuterium exchange rate depends on the solvent accessibility, secondary structure, and structural rigidity (Konermann et al., 2011). Coupled with proteolytic digestion and peptide separation, HDX-MS allows the quantification of exchange rates in short protein segments, up to single-residue resolution. Two major advantages of HDX-MS are that small protein amounts (< 0.1 mg) are required for analysis and that chemical protein labeling is not required, avoiding unwanted structural perturbations. Here, we summarize recent advancements and applications in the use of HDX-MS for studying the structural dynamics of transporters.

## Hydrogen-Deuterium Exchange Mass-Spectrometry Principles

HDX-MS principles are briefly and qualitatively described below, to allow a discussion of its applications for studying transporters. For in-depth explanations, several excellent review articles are available (Konermann et al., 2011; Rand et al., 2014; Engen and Wales, 2015; Masson et al., 2017; Oganesyan et al., 2018; Masson et al., 2019; Redhair et al., 2019).

**Figure 1:**
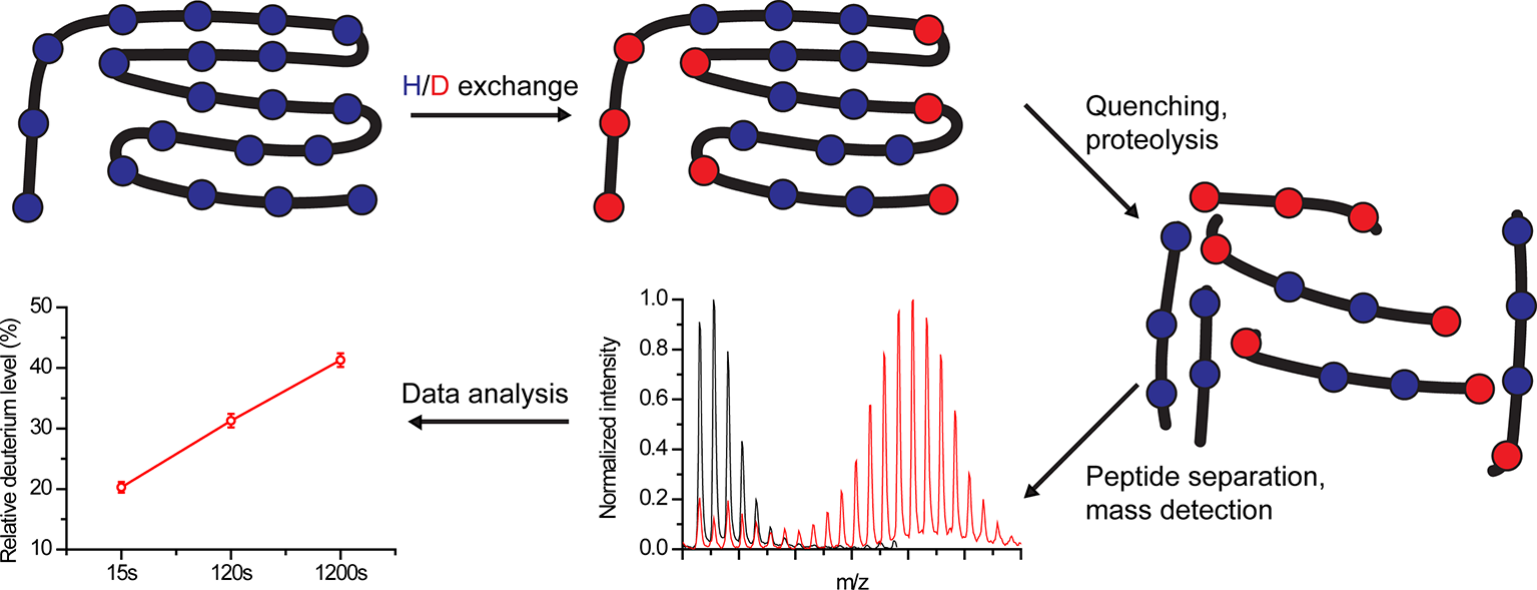
Schematic overview of hydrogen-deuterium exchange mass-spectrometry (HDX-MS) workflow. Proteins are labeled in D_2_O buffer for a predefined time, followed by exchange quenching and proteolysis. The peptides are separated, and their mass is detected using MS. Finally, the amount of incorporated deuterium within each peptide is calculated for each time point.

In HDX experiments, deuterium exchanges with labile protein hydrogens in a time-dependent manner following protein dilution in a D_2_O buffer (Masson et al., 2017) (**Figure 1**). HDX-MS monitors mainly the exchange of backbone amide hydrogens since *i*) at neutral pH they are exchanged in rates that can be detected using HDX-MS (seconds to days) and *ii*) their exchange can be effectively quenched (Marcsisin and Engen, 2010; Gallagher and Hudgens, 2016). The HDX rate depends on several parameters. Amide hydrogens can exhibit a “closed state,” stemming from participation in stable hydrogen bonds in secondary structures or from solvent inaccessibility, which does not allow their exchange with solvent deuterium, or an “open state” where proton abstraction, the rate-limiting step of HDX, can occur (Oganesyan et al., 2018). In addition, the reaction has an intrinsic chemical rate constant, which depends on the pH, temperature, and the residue environment (Oganesyan et al., 2018).

The transition between the closed and open states occurs by local unfolding events. Under native conditions, in which proteins are well folded, the HDX rate mainly depends on the rate of transition back to the closed state (Weis et al., 2006). If the unfolded protein region returns to the closed state at a rate much slower than the chemical exchange rate, EX1 kinetics are observed, resulting in correlated deuterium uptake in neighboring residues. This results in two populations: one with low *m/z* and one with a high *m/z*, reflecting peptides from protein molecules that have not and that have undergone exchange, respectively. With time, the high *m/z* population becomes more prominent at the expense of the low *m/z* population. EX1 kinetics are considered rare in folded proteins under native conditions, but can be induced, for example, by the addition of denaturants (Weis et al., 2006; Oganesyan et al., 2018). If the protein returns to the closed state at a rate much faster than the chemical exchange rate, EX2 kinetics are observed. Here, the exchange depends on the transition of individual residues between the open and closed state, occurring in an uncorrelated fashion. Thus, with time, a single population with gradually increasing *m/z* values is observed (Weis et al., 2006).

Following deuteration, the reaction is quenched at pre-defined time points by changing the pH from neutral (7) to pH_min_ (2.5–3), resulting in ~10,000-fold reduction in HDX (Konermann et al., 2011; Masson et al., 2017; Oganesyan et al., 2018); and by reducing the temperature from 25 to 0°C, leading to ~14-fold decrease in HDX (Englander, 2006; Oganesyan et al., 2018). Next, the protein is digested using a protease and the peptides are separated using analytic reverse-phase chromatography. Currently, the major technical bottleneck of HDX-MS experiments lies in obtaining a high sequence coverage, which is limited by the resistance to digestive enzymes and by proteolytic conditions. Finally, eluted peptides are identified using MS and the degree of HDX is calculated based on the obtained *m/z* values. The experimental details and pitfalls of protein digestion, peptide separation, and MS identification are beyond the scope of this review (Konermann et al., 2011; Masson et al., 2017; Masson et al., 2019).

## Hydrogen-Deuterium Exchange Mass-Spectrometry Studies of Membrane Proteins

Despite sharing the alternating access paradigm, ligand-induced conformational changes differ among transporters due to differences in ligand-protein interactions. For example, symporters (co-transporting two or more ligands) can transition between the IF and OF states without bound ligands, whereas ligand binding is obligatory for swapping between the IF and OF states in antiporters (exchanging ligands at opposite sides of the membrane) (Forrest et al., 2011; Drew and Boudker, 2016; Bai et al., 2017).

In general, detecting local ligand-induced dynamic changes in proteins is difficult. For example, the crystal structures of membrane proteins in both the ligand-free and ligand-bound forms are frequently unavailable, precluding the detection of ligand-induced conformational changes even for proteins with known structures. Moreover, high-resolution structural snapshots of the apo and ligand-bound states may not resolve significant conformational differences. However, small ligand-induced conformational changes can be detected using HDX-MS at specific protein subdomains under native conditions by measuring the differential deuterium uptake (ΔHDX) in the presence and absence of ligands. This ability of HDX-MS provides unique opportunities for addressing the most challenging issues in elucidating the mechanisms of ligand-protein and protein-protein interactions (Rand et al., 2014; Masson et al., 2019; Redhair et al., 2019), as reviewed here for a number of recently studied transporter proteins.

### Conformational Transitions Underlying Alternating Access: The Na^+^/H^+^ Antiporter

Nha proteins regulate cellular pH, [Na^+^], and volume throughout the kingdoms of life (Padan and Landau, 2016). The main structural model used to study Nha orthologs is *Escherichia coli* NhaA, for which a crystallographic structure of the inactive state at acidic pH is available (Hunte et al., 2005). To study the structural dynamics of NhaA under physiological pH, HDX-MS was recently used (Eisinger et al., 2017). Crucial to the insights obtained in this study, HDX-MS provides global dynamic data, in contrast to methods based on site-specific labeling, which only detect movements of pre-defined protein regions.

In this study, an exceptionally high sequence coverage of 88.5% was obtained for NhaA in detergent, providing insights into the mechanism underlying alternating access upon ligand binding. By comparing the HDX patterns between apo- and substrate-bound Nha, a recurring pattern of HDX change was observed in several helices, where increased deuterium uptake in one terminus was accompanied by decreased deuterium uptake at the other terminus. The uptake at the middle of the helices was largely unchanged.

Based on the observed pattern of HDX change, although not directly providing spatial information, it was suggested that translation of the transmembrane helices relative to the membrane occurs, as reflected in the reciprocal HDX changes observed for the two termini within specific transmembrane helices. This model is consistent with the “elevator-like” mechanism of alternating access, implying a vertical translation of transmembrane helices during the transport cycle (Ryan and Vandenberg, 2016). In summary, HDX-MS provided novel insights into the structural transitions involved in alternating access, unattainable by the previously resolved structures of the inactive NhaA.

### Effect of Protein-Lipid Interactions on Transporters' Conformation Landscapes

Most secondary transporters belong to the major facilitator superfamily (MFS), sharing a conserved architecture despite their diverse functions (Radestock and Forrest, 2011). Although the crystal structures of MFS members are available, they provided little information on protein-lipid interactions and their effect on the equilibrium between the OF and IF states. Thus, Martens et al. combined MD simulations with HDX-MS to study the effects of the lipid environment on three well-characterized transporters: lactose permease, xylose transporter, and glycerol-3-phosphate antiporter (Martens et al., 2018).

First, a mutation known to shift the equilibrium toward the OF-state was introduced at the extracellular vestibule of all transporters (Kumar et al., 2014). Comparing the WT and mutated transporters using HDX-MS revealed that the method detects the conformational change, with regions on the extracellular vestibule taking up more deuterium in the mutants *vs.* the WT, whereas the opposite occurs for residues at the intracellular vestibule. Next, the transporters were reconstituted into nanodisks composed of phosphatidylglycerol, tetraoleyl cardiolipin, and either phosphatidylcholine or phosphatidylethanolamine. Interestingly, the presence of phosphatidylethanolamine shifted the equilibrium toward the IF state. Subsequently, MD simulations of the transporters in lipid bilayers identical to the nanodisks' composition predicted direct interactions between the charged phosphatidylethanolamine headgroup and a conserved cytoplasmic network of charged residues, stabilizing the OF state (Doki et al., 2013). Mutating the charged residues abolished the effect of phosphatidylethanolamine, strongly suggesting that specific phosphatidylethanolamine-protein interactions control the intrinsic equilibrium of the transporters. This study is a stark example of combining experimental and computational methods in order to identify subtle but functionally significant protein-lipid interactions.

### Helix Unwinding During Transport: Studies of LeuT

LeuT is a prokaryotic homolog of the neurotransmitter/Na^+^ symporters (NSS) family, which includes important drug targets such as the serotonin, dopamine, and norepinephrine transporters (Kristensen et al., 2011). LeuT was extensively studied, and its crystallographic structures along the transport cycle are available (Focke et al., 2013). These structures suggested that large-scale structural rearrangements are required during alternating access (Krishnamurthy and Gouaux, 2012). However, the conformational landscape allowing the transition between the IF and OF states remains elusive and controversial.

To study the transition between different states, detergent-solubilized LeuT was investigated under conditions that favor the IF or OF states (Merkle et al., 2018). Surprisingly, many peptides, mainly at the intracellular side of the transporter, exhibited EX1 kinetics. This is rather unusual in folded proteins under native conditions and considered to reflect a long-lived unfolding of secondary structure elements. Based on the spatial distribution of peptides exhibiting EX1 kinetics, along with the available crystallographic structures, it was proposed that specific helices undergo partial unwinding during alternating access and that these conformational changes also contribute to substrate release. This study highlights the functional importance of slow (seconds time scale) conformational changes that can be well resolved using HDX-MS, in contrast to other experimental or computational (e.g., MD) methods.

### Hydrogen-Deuterium Exchange Mass-Spectrometry of Membrane Proteins in Lipid Nanodisks

The interactions of membrane proteins with surrounding lipids can dramatically modulate their function (Vadas et al., 2017). Indeed, the activity of eukaryotic NSS members significantly depends on specific lipid-protein interactions that modulate the protein dynamics and consequently, substrate interactions (Divito and Amara, 2009). Therefore, Adhikary et al. studied LeuT reconstituted into phospholipid bilayer nanodisks (Adhikary et al., 2017). Using this approach, LeuT was investigated under conditions that favor the IF and OF conformations. Comparison of the HDX patterns between these states revealed specific alterations in regions previously implicated in the transport cycle, reflecting functionally significant structural changes. Interestingly, the HDX-MS data, consistent with previous biophysical and computational studies, supported a smaller tilt angle for the first transmembrane helix in the IF conformation compared to that observed in the crystal structures. This difference was attributed to the lipid environment, since the crystal structure was obtained in detergent with minute amount of lipid. To summarize, HDX-MS provides a flexible platform to study membrane proteins in different hydrophobic environments and under conditions that favor specific conformational states, without the need for site-specific labeling.

### Ion Interactions With Multiple Sites: The Na^+^/Ca^2+^ Exchanger

NCX participates in cellular Ca^2+^ homeostasis by extruding Ca^2+^ from the cells against its electrochemical gradient (Blaustein and Lederer, 1999). NCX exchanges 3Na^+^:1Ca^2+^, where Na^+^ and Ca^2+^ are transported in separate steps (Khananshvili, 1990). Surprisingly, the crystal structure of NCX from *Methanocaldococcus jannaschii* (NCX_Mj) revealed four ion binding sites, simultaneously occupied by three Na^+^ ions at sites termed S_int_, S_mid_, S_ext_, and one Ca^2+^ ion at a site termed S_Ca_ (Liao et al., 2012). Since this binding mode was inconsistent with previous studies, MD simulations and ion flux analyses of mutants were performed, suggesting that Na^+^ ions occupy S_int_, S_Ca_, and S_ext_, whereas Ca^2+^ occupies S_Ca_ (Marinelli et al., 2014; Giladi et al., 2016b; Giladi et al., 2017; van Dijk et al., 2018). Thus, the Na^+^ and Ca^2+^ ions are bound in a mutually exclusive manner along the transport cycle.

To experimentally establish the assignment of ion binding sites, we compared the apo-state with the ion-bound states of NCX_Mj using HDX-MS (Giladi et al., 2016a; Giladi et al., 2017). Despite low sequence coverage, our analysis included 10 out of 12 ion-coordinating residues. We detected a Na^+^-dependent decrease in deuterium uptake at S_int_, S_Ca_, and S_ext_, but not S_mid_, whereas in the presence of Ca^2+^ the decrease in deuterium uptake was mainly observed at S_Ca_. This is consistent with the predictions made by the MD simulations and mutational analyses foreseeing the occupation of S_mid_ by a water molecule but not by Na^+^ or Ca^2+^ in the ground state (Marinelli et al., 2014; Giladi et al., 2016a; Giladi et al., 2017; van Dijk et al., 2018). Notably, subsequent crystallographic studies have validated our binding sites' assignment (Liao et al., 2016). Thus, HDX-MS corroborated the ion binding mode suggested by the computational and functional studies.

### Ion Selectivity of a Li^+^-Transporting NCX Mutant

The mitochondrial Na^+^/Ca^2+^ exchanger (NCLX) exhibits exceptional ion selectivity, exchanging Ca^2+^ with either Na^+^ or Li^+^, whereas NCXs do not transport Li^+^ (Palty et al., 2010). Although the physiological relevance of this ion selectivity remains puzzling, it is notable that 9 (out of 12) ion-coordinating residues in NCLX differ from those in NCXs and other members of the Ca^2+^/cation antiporter superfamily. To understand how these differences affect the ion binding recognition and transport, we performed structure-based replacement of ion-coordinating residues in NCX_Mj to imitate the NCLX binding sites (Refaeli et al., 2016). Strikingly, the newly designed construct (termed NCLX_Mj) mediates Na^+^/Ca^2+^ and Li^+^/Ca^2+^ with comparable K_m_ values (Refaeli et al., 2016).

Next, we sought to determine whether the ion binding sites in NCLX_Mj are reminiscent of those of NCX_Mj (Giladi et al., 2019). HDX-MS analyses of ion-induced conformational changes revealed that S_Ca_ binds Na^+^, Li^+^, or Ca^2+^, whereas one or more additional Na^+^/Li^+^ sites of NCLX_Mj are incompatible with the original Na^+^ sites (S_ext_ and S_int_) assigned to NCX_Mj. These results suggested that NCLX_Mj may transport ions with an electroneutral stoichiometry of 2Na^+^:1Ca^2+^ or 2Li^+^:1Ca^2+^. Consistent with the HDX-MS data, voltage clamping accelerates the Na^+^/Ca^2+^ exchange rates in NCX_Mj-reconstituted proteoliposomes (due to a stoichiometry of 3Na^+^:1Ca^2+^), whereas it has no appreciable effect on the Na^+^/Ca^2+^ or Li^+^/Ca^2+^ exchange rates in NCLX_Mj (Giladi et al., 2019).

Our studies have demonstrated the utility of HDX-MS in identifying and validating binding sites in ion transporters, where relatively small differences in the ion-induced ΔHDX signals provide important information on ion selectivity and conformational changes occurring upon ion binding at distinct sites. Thus, combined with MD simulations and X-ray crystallography, HDX-MS is especially appealing for elucidating the structural determinants of ion selectivity and ion-induced conformational changes in ion transport systems comprising multiple ion binding sites.

## Conclusions

Over the past decades, structural biology has enormously contributed to our understanding of membrane protein function, mainly by providing static snapshots of discrete states in high resolution. To fully decipher the structure-function relationships underlying the complex process of solute transport, a growing number of experimental and computational methods have been developed to bridge the gap between these static snapshots and determine the underlying conformational landscapes. HDX-MS is increasingly used to study intact membrane proteins under various near-native conditions, providing novel opportunities to study membrane-protein interactions, substrate recognition, and transport-related conformational transitions. Future developments in instrumentation and data analysis automation may allow the use of HDX-MS in high-throughput, fully exploiting its potential use in basic research and biomedical applications such as drug design.

## Author Contributions

MG and DK reviewed the literature and wrote the manuscript.

## Funding

This work was supported by the Israel Science Foundation (Grant #1351/18) (D.K) and by the Israel Cancer Research Foundation (Grant #19202) (MG). Financial support from the Shmuel Shalit award to DK is gratefully acknowledged.

## Conflict of Interest

The authors declare that the research was conducted in the absence of any commercial or financial relationships that could be construed as a potential conflict of interest.
